# Medicinal herb extracts ameliorate impaired growth performance and intestinal lesion of newborn piglets challenged with the virulent porcine epidemic diarrhea virus

**DOI:** 10.1186/s40781-015-0065-1

**Published:** 2015-10-08

**Authors:** Hyeun Bum Kim, Chul Young Lee, Sung Jae Kim, Jeong Hee Han, Keum Hwa Choi

**Affiliations:** Department of Animal Resource and Science, Dankook University, Cheonan, 330-714 South Korea; Regional Animal Industry Center, Gyeongnam National University of Science and Technology, Jinju, 660-758 South Korea; Department of Veterinary Pathology, College of Veterinary Medicine and Institute of Veterinary Science, Kangwon National University, Chuncheon, 200-701 South Korea; Department of Complementary and Alternative Medicine, College of Veterinary Medicine, University of Minnesota, Saint Paul, 55108 USA

**Keywords:** Health, Medicinal herb, Pigs, Porcine epidemic diarrhea

## Abstract

The objective of this study was to evaluate effects of a combined use of extracts of medicinal herbs *Taraxaumi mongolicum, Viola yedoensis Makino, Rhizoma coptidis,* and *Radix isatidis* (MYCI) on porcine epidemic diarrhea (PED). Twenty-two 3-day-old piglets received an oral challenge with 3 × 10^3.5^ TCID_50_ of the virulent PED virus (PEDV) in PBS or PBS only and daily oral administration of 60 mg of the MYCI mixture suspended in milk replacer or the vehicle for 7 days in a 2 × 2 factorial arrangement of treatments. Average daily gain (ADG) increased (*p* < 0.05) in response to the MYCI treatment in the PEDV-challenged piglets (−18 vs. 7 g for the vehicle- vs. MYCI-administered group), but not in unchallenged animals (27 vs. 28 g). Diarrhea score and fecal PEDV shedding, however, were not influenced by the MYCI treatment. The PEDV challenge caused severe intestinal villus atrophy and crypt hyperplasia, both of which were alleviated by administration of the MYCI mixture as indicated by an increase in the villus height and a decrease in the crypt depth due to the treatment. Overall, medicinal herb extracts used in this study ameliorated impaired growth performance and intestinal lesion of newborn piglets challenged with the virulent PEDV. Therefore, our results suggest that the MYCI mixture could be used as a prophylactic or therapeutic agent against PED.

## Background

The porcine epidemic diarrhea virus (PEDV) or genus *Alphacoronavirus* causes an acute or sub-acute gastrointestinal disorder in pigs of all ages resulting in serious economic losses across Europe and Asia [1–3]. Primary clinical symptoms of the PEDV infection include acute diarrhea, vomiting, weight loss, and dehydration [4, 5]. Moreover, an increased mortality as well as higher morbidity in suckling piglets vs. older ones has also been reported as to the damage of PEDV infection [6]. Many attempts have been made to find effective prophylactic or therapeutic agents against PEDV infection, but no satisfactory agent has been reported to date [7, 8].

Medicinal herbs have been used as antimicrobial agents for many years in Asian countries [9–12]. For example, extracts of *Houttuynia cordata* are known to be effective against herpes simplex virus type 1 (HSV-1), influenza virus, human immunodeficiency virus type 1 (HIV-1) [13], and coronavirus [14]. Moreover, *Taraxacum mongolicum, Viola yedoensis Makino, Rhizoma coptidis,* and *Radix isatidis,* which have been selected for the present study, are also known to have antimicrobial and antiviral properties [15]. *Taraxacum mongolicum* has anti-inflammatory, anti-carcinogenic, anti-allergic and anti-viral properties [16–18] whereas extracts of *Viola yedoensis Makino* have antiviral and antibacterial activities against HSV-1 and *Bacillus subtilis* [19, 20]. *Rhizoma coptidis*, which contains berberine as one of its active components, has a potent antiviral activity against herpes virus as well as an antibacterial activity [21, 22]. *Radix isatidis,* roots and leaves of which have been used in traditional Eastern medicine for centuries to treat upper respiratory infections and encephalitis, has been reported to have anti-inflammatory [23] and antiviral effects against hepatitis, severe acute respiratory syndrome (SARS), influenza, and epidemic encephalitis [24] as well as a stimulatory action on macrophage phagocytosis [25].

*Taraxacum mongolicum, Radix isatidis, Viola yedoensis Makino, Rhizoma coptidis,* and *Radix isatidis* are potentially useful medicinal herbs for PED as could be extrapolated from their antimicrobial activities described above. To our knowledge, however, such a possibility has not been examined to date. The present study was therefore conducted to investigate the effects of a combined mixture of extracts of the four herbs (MYCI) on growth performance, diarrhea, and intestinal morphology in newborn piglets challenged with PEDV.

## Methods

### Animals and treatments

The experimental protocol regarding animal management and care was approved by the Animal Care and Use Committee of Kangwon National University. The MYCI reagent used in the present study was prepared by mixing powdered extracts of *Taraxaci mongolia, Viola yedoensis Makino, Rhizoma coptidis* and *Radix isatidis* (Mayway Co., USA) at a ratio of 1:1:1:2. Twenty-two 3-day-old cross-bred [(Landrace × Yorkshire) × Duroc] suckling piglets were obtained from a commercial farm that had not implemented PEDV vaccination. The piglets were housed individually and randomly assigned to one of four experimental groups in a 2 × 2 factorial arrangement of treatments with respect to a PEDV challenge and administration of the MYCI mixture. The animals received an oral administration of 3 × 10^3.5^ TCID_50_ of PEDV in 3 mL of phosphate-buffered saline (PBS) or PBS only following 12-h fasting and daily administration of 60 mg of the MYCI mixture suspended in 1 mL of milk replacer (CJ Feed, Inc., Seoul) or the vehicle for 7 days. Calculated chemical composition of the milk replacer is presented in Table 1. All animals had a free access to water and the milk replacer throughout the experiment. Feces was obtained directly from the rectum of each piglet at 12 and 24 h and daily after d 1 post-challenge. Fecal consistency was scored according to a 4-ladder scale as described previously [26]: 0, firm feces; 1, soft feces; 2, mild diarrhea; 3, severe diarrhea. All animals were killed by electric stunning upon termination of the feeding trial, and the duodenum, jejunum, and ileum were dissected also as described [26].

**Table 1:** Calculated chemical composition of the milk replacer

Item	Value
Crude protein (%)	20.5
Crede fat (%)	15.0
Crude fiber (%)	3.0
Crude ash (%)	11.0
Calcium (%)	0.8
Phosphorous (%)	1.5
Vitamin A (U/kg)	40,000

### Detection of fecal shedding of PEDV

RNA contained in the feces was extracted using RNasy Mini Kit® (QIAGN, Germany) after removal of contaminants by centrifugation for 10 min at 4000 × *g*. Presence of PEDV in the sample was identified by the reverse transcription-polymerase chain reaction (RT-PCR) targeting a coding region of the membrane protein of the PEDV [14]. The base sequences of the forward and reverse primers were 5’-GGGCGCCTGTATAGAGTTTA-3’ and 5’-AGACCACCAAGAATGTGTCC-3’, respectively. The PCR conditions were initial denaturation at 95 °C for 2 min, 35 cycles of 95 °C for 20 s, 56 °C for 40 s, and 72 °C for 1 min, and final extension at 72 °C for 3 min. A 412-bp PCR product was identified by the agarose gel electrophoresis.

### Histopathological examination

The intestinal tissue was fixed in a 10 % neutral formalin solution. The fixed tissue was embedded in paraffin, sectioned to a 4-μm thickness, stained with hematoxylin and eosin, and subjected to measurement of the villus height (VH) and crypt depth (CD) under a microscopic field as previously described [26].

### Statistical analysis

Data were analyzed using the GLM procedure of SAS (SAS Inst., USA) in all variables except for fecal PEDV shedding. The statistical model included the PEDV challenge and administration of the MYCI mixture as main effects as well as their interaction. In the frequency of fecal PEDV shedding, which was analyzed using the chi-squared test of SAS, effects of the PEDV challenge and administration of the MYCI mixture were analyzed separately.

## Results

### Growth performance

The initial and final weights of the newborn piglets did not differ between the PEDV-challenged and unchallenged piglets or between the MYCI mixture-administered piglets and the vehicle (milk replacer)-administered (control) ones (Table 2). However, average daily gain (ADG) during the 7-d experimental period decreased (*p* < 0.01) due to the challenge (27 vs. −6 g for the unchallenged vs. challenged group). The ADG increased (*p* < 0.05) in response to administration of the MYCI mixture (17 g vs. 4 g for the MYCI vs. control group). Moreover, ADG increased due to administration of the MYCI mixture in the challenged piglets, but not in the unchallenged ones.

**Table 2:** Effects of oral administration of the MYCI mixture on growth performance of newborn piglets after an oral administration of PEDV^a)^

	Not challenged			Challenged (Chal)			*P*-value		
	CON^b)^ (*n* = 5)	MYCI (*n* = 5)	SEM	CON (*n* = 6)	MYCI (*n* = 6)	SEM	Chal	MYCI	C × M
Initial wt, kg	2.61	2.69	0.251	2.55	2.60	0.229	0.758	0.801	0.946
Final wt, kg	2.80	2.89	0.258	2.43	2.65	0.235	0.232	0.545	0.797
ADG^b)^, g	27	28	5.1	−18	7	4.7	<0.001	0.016	0.029

^a^Newborn piglets received an oral challenge with 3 × 10^3.5^ TCID_50_ of PEDV in PBS or PBS only and daily administration 60 mg of the MYCI mixture suspended in milk replacer or the vehicle for 7 days

^b^CON, control; ADG, average daily gain

### Diarrhea and detection of fecal shedding of PEDV

**Fig. 1:**
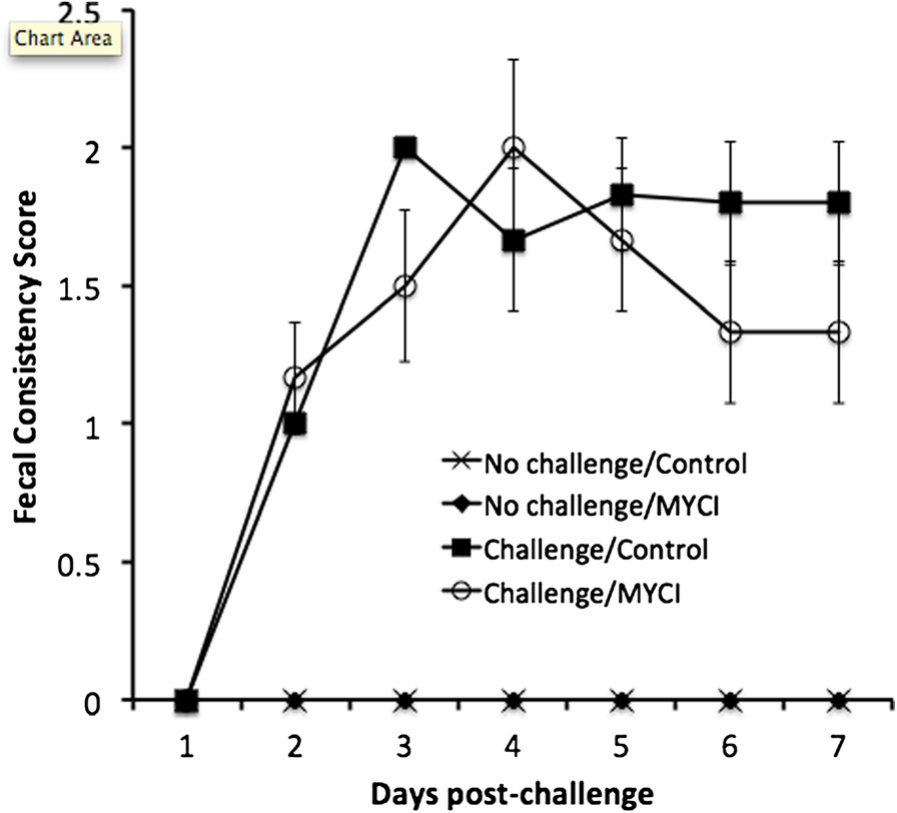
Effects of the oral PEDV challenge and administration of the MYCI mixture on fecal consistency score of newborn piglets. Newborn piglets received a challenge with 3 × 10^3.5^TCID_50_ of PEDV in PBS or PBS only and daily administration of 60 mg of the MYCI mixture suspended in milk replacer or the vehicle. Rectal feces was scored according to a 4-ladder scale: 0, firm feces; 1, soft feces; 2, mild diarrhea; 3, severe diarrhea. The effect of the PEDV challenge was significant (*p* < 0.05)

**Fig. 2:**
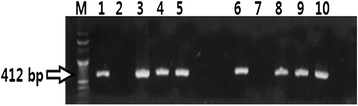
PED virus detection in the small intestine and fecal samples using RT-PCR assay. Lane M; 100 bp DNA ladder. Lanes 1 and 6; a 412-bp PCR product (membrane protein of the PED virus; a positive control). Lanes 2 and 7; negative controls. Lanes 3–5; small intestine samples of the PED virus challenged piglets on d 7. Lanes 8–10; fecal samples of the PED virus challenged piglets on d 7

The piglets challenged with PEDV exhibited watery diarrhea beginning from d 2 post-challenge while unchallenged piglets had normal firm feces throughout the experiment (Figure 1). Administration of the MYCI mixture had no effect on the fecal consistency score. The presence of PED virus in the feces and in the small intestines was examined by RT-PCR (Table 3 and Fig. 2). When the virus was detected, it was present in both feces and small intestine (Fig. 2). The PEDV was detected in feces from d 1 through the end of the experiment only in the challenged piglets (Table 3). Moreover, the effect of the challenge on the frequency of the fecal PEDV shedding on d 1 onwards was significant (*p* < 0.05) whereas the effect of the MYCI administration was not significant at any time point of the experiment.

**Table 3:** Effects of oral administration of the MYCI mixture on fecal PEDV shedding of newborn piglets after an oral administration of the virus^a)^

Time	Not challenged		Challenged (Chal)		*p*-value	
	Control (*n* = 5)	MYCI (*n* = 5)	Control (*n* = 6)	MYCI (*n* = 6)	Chal	MYCI
0 h	0/5	0/5	0/6	0/6	N/A^b^	N/A
12 h	0/5	0/5	0/6	0/6	N/A	N/A
1 d	0/5	0/5	2/6	2/6	0.044	1.000
2 d	0/5	0/5	6/6	6/6	<0.001	1.000
3d	0/5	0/5	6/6	5/6	<0.001	0.670
4 d	0/5	0/5	4/6	2/6	0.009	0.338
5 d	0/5	0/5	4/6	3/6	0.003	0.647
6 d	0/5	0/5	3/5^c^	3/6	0.006	0.890
7 d	0/5	0/5	2/5	2/6	0.034	0.916
Total	0/45	0/45	27/52	23/54	<0.001	0.460

^a^Newborn piglets received an oral challenge with 3 × 10^3.5^ TCID_50_ of PEDV in PBS or PBS only and daily administration 60 mg of the MYCI mixture suspended in milk replacer or the vehicle for 7 days. Presence of the PEDV in feces was determined by reverse transcription-polymerase chain reaction. Data represent the number of PEDV-positive animals per total animals

^b^N/A, Not applicable

^c^One animal died

### Intestinal morphology

**Fig. 3:**
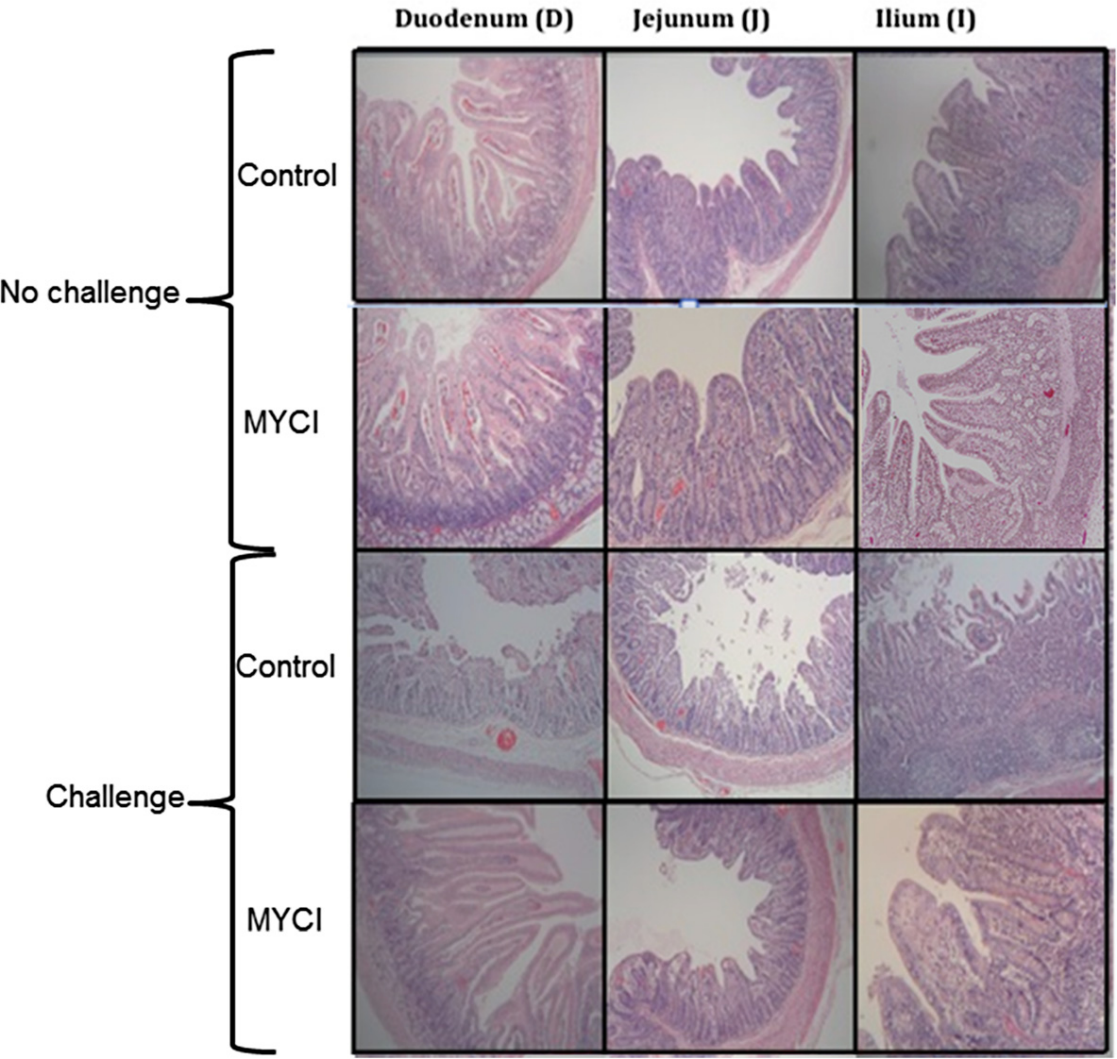
Effects of the oral PEDV challenge and administration of the MYCI mixture on intestinal morphology of newborn piglets (100× magnification). Newborn piglets received a challenge with 3 × 10^3.5^TCID_50_ of PEDV in PBS or PBS only and daily administration of 60 mg of the MYCI mixture suspended in milk replacer or the vehicle for 7 days. Note the atrophied and fused villi as well as crypt hyperplasia in the challenged/control group and the alleviation of the atrophy and fusion of the villi and the crypt hyperplasia in the challenged/MYCI group compared with the morphology in the former. Only representative results are shown in this figure

The PEDV-challenged piglets exhibited a severe atrophy in the villus structure as well as hyperplasia with infiltration of inflammatory cells in the crypt in the duodenum, jejunum, and ileum when their intestinal morphology was compared with that of the unchallenged piglets (Fig. 3). The villus atrophy and crypt hyperplasia due to the PEDV infection were alleviated when the MYCI mixture was administered to the challenged piglets.

The VH in the duodenum decreased due to the PEDV challenge, but it increased in response to administration of the MYCI mixture (Table 4). Duodenal CD, however, was not influenced by the challenge or administration of the MYCI mixture. The VH:CD ratio in the duodenum was greater in the MYCI mixture-administered group vs. the control only in the animals challenged with PEDV. In the Jejunum, VH, which did not change due to the challenge, increased in response to administration of the MYCI mixture. The CD in the jejunum increased due to the challenge. Moreover, CD in this region decreased in response to administration of the MYCI mixture, which was apparent only in the PEDV-challenged animals. The VH in the ileum also increased due to administration of the MYCI mixture, which was apparent only in the PEDV-challenged animals. The CD in the ileum decreased due to the PEDV challenge as well as administration of the MYCI mixture.

**Table 4:** Effects of oral administration of the MYCI mixture on intestinal mucosal morphology of newborn piglets after an oral administration of PEDV^a)^

	Not challenged			Challenged (Chal)			*P*-value		
	CON^b)^ (*n* = 5)	MYCI (*n* = 5)	SEM	CON (*n* = 6)	MYCI (*n* = 6)	SEM	Chal	MYCI	C × M
Duodenum									
VH^b)^, μm	443	482	38.3	409	471	31.7	0.050	<0.001	0.023
CD^b)^, μm	171	171	10.7	183	173	16.1	0.183	0.255	0.533
VH:CD	2.65	2.72	0.163	2.31	2.74	0.149	0.590	0.392	0.002
Jejunum									
VH, μm	144	151	11.7	129	154	13.3	0.156	0.002	0.030
CD, μm	157	137	13.0	170	138	9.5	0.040	<0.001	0.001
VH:CD	0.98	1.18	0.166	0.89	1.23	0.155	0.946	0.377	0.013
Ilium									
VH, μm	166	182	13.3	161	184	10.1	0.648	<0.001	0.015
CD, μm	165	166	15.9	160	142	11.0	0.001	0.027	0.048
VH:CD	1.12	1.16	0.156	1.01	1.32	0.170	0.933	0.547	0.058

^a^Newborn piglets received an oral challenge with 3 × 10^3.5^ TCID_50_ of PEDV in PBS or PBS only and daily administration 60 mg of the MYCI mixture suspended in milk replacer or the vehicle for 7 days

^b^CON, control; VH, villus height; CD, crypt depth

## Discussion

While only sporadic PED outbreaks were reported in Europe during the past few decades, PEDV infection has caused enormous economic losses in the pig industry in Asian countries including China, South Korea and Vietnam during the same period. Furthermore, swine farmers in North America have also been experiencing devastating economic losses resulting from prevalence of PED since its outbreak in April of 2013 in the USA [27]. Because the efficacy of PEDV vaccines is not good enough to prevent PEDV infection, it has been a research focus to search for safe, inexpensive and effective measures to prevent or treat PED. Accordingly, a variety of studies including the present one have been conducted in Europe, Asia, and America to find antiviral agents in medicinal herbs as a means of alternative medicine [6, 28].

Diarrhea and vomiting are the main clinical signs of PEDV infection resulting in suboptimal growth performance in suckling piglets [29]. The decreased ADG as well as diarrhea of the newborn piglets following the PEDV challenge in the present study was thus consistent with the known clinical signs of the virus infection [29, 30]. More importantly, administration of the MYCI mixture alleviated the growth check of the piglets due to the PEDV infection.

The PEDV infection causes gross and microscopic lesions such as severe atrophic enteritis with villous fusion similar to those found in the infection of the transmissible gastroenteritis virus [29, 31]. The atrophied villus structure as well as crypt hyperplasia indicated by the decreased VH and increased CD observed in the PEDV-challenged piglets was therefore consistent with the known histopathological signs of the PEDV infection. Furthermore, the increase in VH in the duodenum, jejunum, and ileum as well as the decrease in CD in the jejunum and ileum in response to administration of the MYCI mixture indicates that the MYCI recipe has a beneficial effect on the integrity of the intestinal mucosal structure of the piglets infected with PEDV.

The ultimate goal of PEDV vaccination is to prevent PEDV infection. However, currently available PEDV vaccines are not effective enough to prevent PED, although some of the vaccines are effective for alleviating fecal virus shedding and the severity of the clinical signs of PED [6]. In this regard, administration of the MYCI mixture was not effective for reducing the frequency of fecal PEDV shedding in the challenged piglets with all its positive effects on the integrity of the intestinal mucosal structure. Nevertheless, it is thought to be worth trying to examine if a combined use of a PEDV vaccine and the MYCI mixture has any synergistic prophylactic or therapeutic effect in PED.

A number of phytochemicals contained in medicinal plants, which include limonoids, alkaloids, lignana, organosulfur, furyl, thiophenes, polylines, terpenoids, flavonoids, polyphenolics, sulphides, saponins, coumarins, and chlorophyllins, are known to exhibit the antiviral effects by blocking viral entry or RNA/DNA replication or by virtue of their anti-oxidant activity [32]. Likewise, active antiviral phytochemicals, i.e. 11β,13-dihydrotaraxinic acid, 6,7-dimethoxycoumarin, berberine, and indirubin, have been identified in *Taraxaumi mongolicum* [33], 6,7- *Viola yedoensis Makino* [34], *Rhizoma coptidis* [21], and *Radix isatidis* [35], respectively, extracts of which were used in the present study. It also seems possible that there exist more active phytochemicals other than those described above in those herb extracts used in the present study, because the list of active components contained in medicinal herb extracts is rapidly expanding [36]. Overall, the present results suggest that administration of the MYCI mixture is a promising recipe which could be used as a prophylactic or therapeutic measure for PED.

## Conclusions

We evaluated effects of a combined use of extracts of medicinal herbs *Taraxaumi mongolicum, Viola yedoensis Makino, Rhizoma coptidis,* and *Radix isatidis* (MYCI) on PED. In summary, medicinal herb extracts used in this study ameliorated impaired growth performance and intestinal lesion of newborn piglets challenged with the virulent PEDV. Therefore, our results suggest that the MYCI mixture could be used as a prophylactic or therapeutic agent in PED.
